# Supplementary material for: Identification of the preoperative and perioperative factors that predict postoperative endothelial cell density after Descemet membrane endothelial keratoplasty: A retrospective cohort study
Source: PLoS One. 2022 Feb 24;17(2):e0264401. doi: 10.1371/journal.pone.0264401 (PMC8870504; doi:10.1371/journal.pone.0264401)
Supplement: S1 Table — (DOCX) [file pone.0264401.s001.docx]

Supplementary Table S1. Summary of the published studies that comprehensively examined a range of pre/perioperative variables for their ability to predict endothelial cell density/loss after DMEK surgery.

| **Variable** | **Rodriguez 2016 [43]**  **Logistic regression**  **Relationship with ECL at 6 mo**  **Mixed indic.**  **N=500** | **Peraza 2017 [20]**  **Linear mixed model**  **Relationship with ECD at 24 mo**  **Mixed indic.**  **N=500** | **Oellerich 2017 [44]**  **Linear mixed model**  **Relationship with ECL at 6 mo**  **Mixed indic.**  **N=2485** | **Oellerich 2020 [45]**  **Whole cohort multiple regression**  **Relationship with ECL at 48 mo**  **Mixed indic.**  **N=351** | **Hayashi 2020 [46]**  **Multivariate regression**  **Relationship with ECL at 12 mo**  **FECD**  **N=841** | **Inoda 2020 [47]**  **Multiple regression**  **Relationship with ECL at 12 mo**  **BK**  **N=72** | **Lekhanont 2021 [48]**  **Linear regression**  **Relationship with ECD at 24 mo**  **Mixed indic.**  **N=62** | **Our study 2022**  **Linear regression**  **Relationship with ECD at 6 and 12 mo**  **FECD**  **N=103** |
| --- | --- | --- | --- | --- | --- | --- | --- | --- |
| **Patient age** | NS | 0.02↓older | NS | 0.049↑older | NS | NS | NS | NS |
| Patient sex | NS | NS | NS | NS | NS | NS | NS | NS |
| **Indication/High FECD grade** | NS | FECD better vs BK 0.01 | NS | Advanced FECD <0.01 |  |  | NS |  |
| Preop VA |  | NS | NS |  |  | NS |  | NS |
| Preop CCT |  | NS |  | NS |  | NS |  |  |
| **Preop lens status** | NS | phakic better vs pseudo 0.007 | NS | NS |  |  |  |  |
| Preop axial length |  |  |  |  |  | NS | NS |  |
| Mean anterior kerat. |  |  |  |  |  |  |  | NS |
| Donor age | NS |  |  | NS | NS |  | NS | NS |
| Donor sex | NS |  |  | NS | NS |  |  |  |
| **Donor ECD** |  |  |  | ? |  | 0.042 | NS | 0.03 univar 6 mo |
| Gr. storage (cold v organ) |  |  | NS |  | NS |  |  |  |
| **Graft storage time** | p=0.01 | NS |  | NS |  |  |  |  |
| **Graft diameter/size, Ratio graft:cornea area** |  |  | NS |  |  | 0.0061 | 0.015 |  |
| Triple vs single DMEK |  |  | NS |  |  |  | NS | NS |
| Air vs SF6 tamponade |  |  |  |  | NS | NS |  |  |
| % air fill |  |  | NS |  |  | NS |  |  |
| **Surgery time** |  |  |  |  |  |  |  | 0.03 univar. 12 mo |
| **Intraoperative complications** |  |  | NS | 0.032* |  |  |  | 0.010 6 mo 0.02 12 mo |
| **Graft attachment (<1/3 v attached)/rebubbling** |  | <0.0001 |  | 0.0002 | F = 16.8 <0.001 | NS | NS | NS |
| **Other postop complic.** |  |  |  | 0.048† |  |  |  |  |

*Problems inserting, unfolding, and /or positioning graft; failure to unfold; inverted graft; intraoperative hemorrhage, other complications including increased posterior vitreous pressure and Descemet membrane remnants

†Allograft rejection, IOP elevation
