# Supplementary material for: Identification of the preoperative and perioperative factors that predict postoperative endothelial cell density after Descemet membrane endothelial keratoplasty: A retrospective cohort study
Source: PLoS One. 2022 Feb 24;17(2):e0264401. doi: 10.1371/journal.pone.0264401 (PMC8870504; doi:10.1371/journal.pone.0264401)
Supplement: Attachment — Submitted filename: renamed_86e1b.docx [file pone.0264401.s002.docx]

Authors describe the Factors that predict postoperative endothelial cell density after Descemet membrane endothelial keratoplasty. Although it’s an interesting study, there’re many points that should be addressed.

This may reflect endothelial cell loss due to excessive graft handling and/or an intrinsic unhealthiness of the endothelial cells in the graft that conferred unwanted physical properties onto the graft that complicated its preparation/unfolding.

1) Authors should discuss after dividing into the factors of graft, and those of patients, which means DMEK for eyes after vitrectomy, or with high myopia, or more. The method that authors defined include many confounding factors. At least, authors should be combine or discuss these two factors of difficult graft preparation or unfolding simultaneously. (Line 37, 182)
2) Please let readers know the definition of the surgical time. Do you mean the time from the starting of graft preparation by surgeons, or the patient’s procedure? Does it include the graft preparation time? If so, your surgical time should be diminished by the duration time of graft preparation. Do you use pre-stripped tissues by eyebanks, or non-stripped tissues?
3) Regarding regression analysis, there is no description of R value.
4) The evaluating factors of simple, and multiple regression analysis seem to be same. How did you decide these evaluating factors with evidence? Authors should consult the statistician.
5) Difficult surgery might be the confounding factor of surgery time. Please consider getting rid of confounding factors from them.
6) “We then divided both the 6- and 12-month cohorts into two subgroups by applying a postoperative ECD cut-off of 1000 cells/mm2. This value was selected because it is considered a risk threshold of chronic corneal edema [Line 271-172]” Eyes with low ECD about 1000 have no corneal edema. Please let me know the reason why you decide the ECD of 1000 was the cut-off point clearly.
7) Please consider showing the dataset.
